# Food knowledge level among Tanzanian women of childbearing age: developing a score for the food knowledge questionnaire

**DOI:** 10.1017/jns.2023.28

**Published:** 2023-04-04

**Authors:** Maria Vittoria Conti, Marco Gnesi, Naelijwa Mshanga, Rachele De Giuseppe, Francesca Giampieri, Hellas Cena

**Affiliations:** 1 Laboratory of Dietetics and Clinical Nutrition, Department of Public Health, Experimental and Forensic Medicine, University of Pavia, Via Bassi 21, Pavia 27100, Italy; 2 Section of Biostatistics and Clinical Epidemiology, Department of Public Health, Experimental and Forensic Medicine, University of Pavia, Pavia 27100, Italy; 3 The Nelson Mandela African Institution of Science and Technology, Arusha 447, Tanzania; 4 Department of Biochemistry, Faculty of Sciences, King Abdulaziz University, Jeddah 21589, Saudi Arabia; 5 Research Group on Food, Nutritional Biochemistry and Health, Universidad Europea del Atlantico, Santander 39011, Spain; 6 Clinical Nutrition and Dietetics Service, Unit of Internal Medicine and Endocrinology, ICS Maugeri IRCCS, Pavia 27100, Italy

**Keywords:** Childbearing age, Developing countries, Food knowledge, Women

## Abstract

Food knowledge (FK) is one of the factors that contribute to malnutrition conditions in developing countries, together with food safety, food security and food access. FK is defined as ‘the competence to understand healthy nutrition concepts’; it impacts individuals’ life due to its relationship with food behaviour and eating habits. Therefore, acting on FK can represent a starting point for improving the health status of vulnerable populations. The authors present a total score of an FK questionnaire (FKQ) and its relation to the socio-demographic characteristics of a specific target population: Tanzanian women of childbearing age. The results of the manuscript complement evidence of construct validity of the FKQ by providing an algorithm to compute a total score as a measure of FK. The strength of this tool, and its score, lies in the fact that the questionnaire has been validated and is easy to administer.

## Introduction

Malnutrition refers to deficiencies, excesses or imbalances in an individual's intake of energy or nutrients^(1)^. The term malnutrition covers two broad groups of conditions: on the one hand, ‘undernutrition’ and micronutrient deficiencies or insufficiencies; on the other hand, overweight, obesity and or other non-communicable diseases (NCDs)^(1)^. Lancet's series on ‘dynamics of the double burden of malnutrition and the changing nutrition reality’ in 2020 suggests that the global health community has been slow in acknowledging and responding to the high prevalence of the double burden of malnutrition, particularly in low- and middle-income countries^(2)^. No area in the world has been exempted from the rising prevalence of overweight and obesity; this has translated into the current obesity pandemic, including a rapid rise in overweight/obesity in sub-Saharan Africa (SSA), with a slower decrease in undernutrition prevalence^(3)^. In Tanzania, 32 % of children under 5 years old are stunted (have low height-for-age), 58 % suffer from anaemia^(4)^ as well as about 40 % of women of childbearing age (14–49 years)^(5)^.

At the same time, 15⋅2 % of adult women (aged 18 years and over) and 5⋅0 % of adult men are affected by obesity^(5)^, with a worsening trend in diabetes and hypertension^(6)^.

All forms of malnutrition may lead to health problems, in particular when vulnerable populations are affected; in childhood and pregnancy, many adverse consequences have been observed as far as child survival and long-term well-being^(3)^ are concerned, with a negative impact on human capital, economic productivity and national development overall^(3)^.

In all its forms, malnutrition still poses a significant health burden in Tanzania: this is influenced not only by the inadequate or excessive intake of energy, macro- and micronutrients, but also by many individual and environmental factors, including lack of nutrition and food knowledge (FK)^(7)^. It has been demonstrated that FK, defined as ‘the competence to understand healthy nutrition concepts’, impacts individuals’ life due to its relationship with food behaviour and eating habits^(8)^.

Positive associations between maternal FK and child nutritional outcomes are well-documented^(9–11)^. It has been shown that, when women of childbearing age receive nutritional education, they exert a positive effect on dietary knowledge and practices, with a positive impact on maternal and offspring health^(12)^.

In this context, assessing food knowledge of women of childbearing age appears essential to the implementation of tailored nutrition education programmes aimed at improving lifestyle and health to redirect food choices and consumption habits^(13)^. In order to come up with a valid and representative result of the context, it is important to use a tool that has been validated for this specific use in the target population, according to a standard methodology^(14)^. In light of this scenario, a food knowledge questionnaire (FKQ) has been previously developed to assess knowledge of various topics related to the role of food connected to health, among Tanzanian women of childbearing age. Evidence supporting construct validity of the FKQ has been presented in another publication^(7)^, disclosing different sections each one related to a different aspect of FK; a user of the FKQ, therefore, had to handle different section scores, each one specific of an aspect of FK, losing a general and comprehensive view of the subject's FK.

In order to provide users with a general measure of FK, the present article introduces a total score obtained from the FKQ and investigates the properties of such score and its relation to the socio-demographic characteristics of Tanzanian women of childbearing age.

## Materials and methods

The FKQ comprises eighty-eight questions, grouped into ten different sections; each section was designed to investigate a different aspect of FK, apart from Section A, which collected social and demographic information^(7,12)^.

The questionnaire has been validated in a previously reported cross-sectional study^(7)^; the sample included 671 women of childbearing age (14–49 years), enrolled from August to October 2020 in the Arusha region and in the Morongoro region (Tanzania). The recruitment was conducted both in urban areas (cities of Arusha – the third major urban centre of the country – and Morogoro) and in peri-urban ones (defined as areas of transition from strictly rural to urban).

Nominal variables were described as frequencies and percentages; pseudo-quantitative variables (questionnaire scores) were summarised by their medians and interquartile ranges (IQRs). Differences in scores between groups were tested by using Mann–Whitney's *U* test (two groups) or Kruskal–Wallis test (three or more groups) with appropriate *post-hoc* tests when applicable. Association between nominal variables was tested with Pearson's χ^2^ test for independence. The significance threshold was set at 0⋅05; Bonferroni correction for multiple testing was applied in *post-hoc* testing. Multiple correspondence analysis (MCA) with Burt's method was used to explore reciprocal relationships between sets of nominal variables.

The total score was computed by counting the sections in which a given respondent scored above or equal to the median score of that section among all respondents: specifically, respondents were assigned a ‘1’ in each section where they were above (or equal) the median score in the entire sample, and a ‘0’ otherwise. Such a computation algorithm flattens the weights of sections’ scores in determining the total score: since each section includes a different number of items, a summative criterion for the total score would give different weights to different sections, making the total score mostly dependent on those sections with the highest number of items.

Statistical analyses were performed in Stata 13⋅1^(15)^.

## Results

**Fig. 1.**
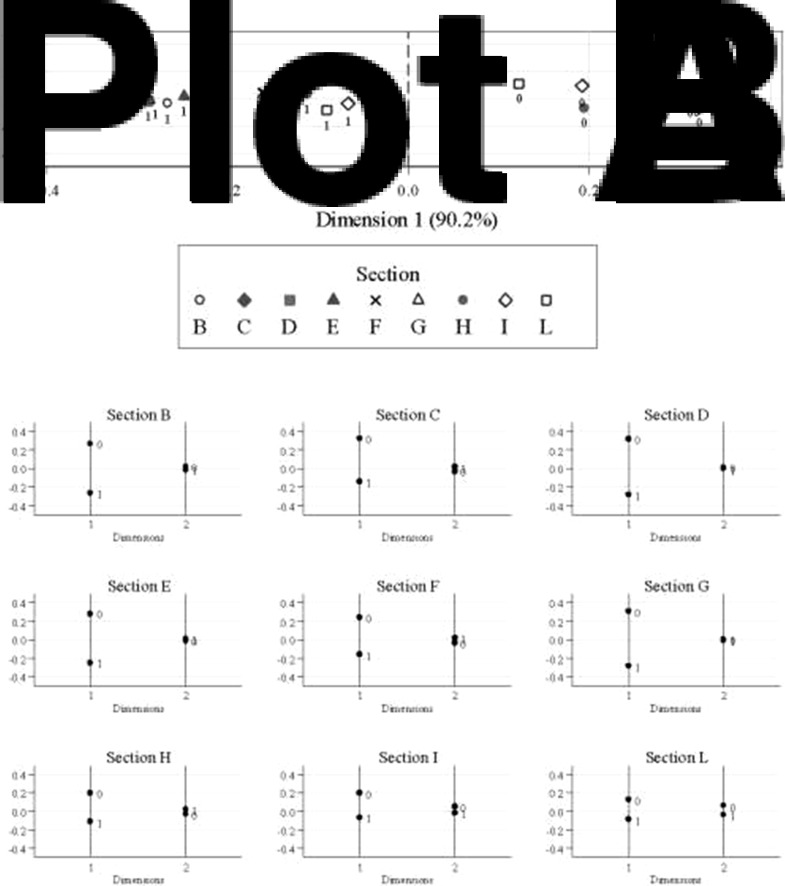
Coordinates plot (Plot A) and projections plot (Plot B) of the multiple correspondence analysis on dichotomised sections’ scores. Dimension 1 (horizontal axis in Plot A) explains 90⋅2 % of inertia, i.e. observed variability. Coordinates are reported in principal normalisation.

A detailed description of sections’ scores can be found elsewhere, together with a description of socio-demographic variables^(7)^. A summary description of sections’ scores is reported in Table 1. In each section, the median score among respondents was used to dichotomise scoring as ‘above median’ or ‘below median’ (Table 1). MCA of dichotomised sections’ scores (Fig. 1) disclosed a single dimension distinguishing between a profile of ‘High FK’ (corresponding to an archetypal subject with all scores above median) and another of ‘Poor FK’ (with all scores below median).

**Table 1.** Descriptive statistics of sections’ scores. *N* 671

Section	Median (95 % CI)	IQR	Min–Max	*n* (%) above median
B	5 (4–5)	2	0–6	337 (50⋅2)
C	2 (2–2)	1	0–3	466 (69⋅5)
D	3 (2–3)	3	0–4	353 (52⋅6)
E	4 (3–4)	2	0–4	358 (53⋅4)
F	3 (3–3)	2	0–4	401 (59⋅8)
G	5 (4–5)	3	0–6	346 (51⋅6)
H	3 (3–3)	1	0–3	426 (63⋅5)
I	2 (2–2)	2	0–3	499 (74⋅4)
L	6 (6–7)	4	0–10	387 (57⋅7)

95 % CI: Binomial exact confidence interval of the median, confidence level 95 %.

Dichotomised sections’ scores were used to compute a total FKQ score as described in the Methods. Such a total score, ranging from 0 to 9, had a median value of 5 (IQR 3); its distribution is represented in Supplementary Figure S1.

The total FKQ score was significantly associated with the level of education and job of the respondent; the score increased with school level and was higher in business-related jobs (Table 2). It is worth noticing that education and jobs were associated themselves (χ^2^ = 38⋅53, *P* < 0⋅001), with better jobs related to higher education (school level). The total FKQ score was also associated with being on a diet, with the scores’ distribution being slightly higher among those who were not on a diet (Table 2).

**Table 2.** Association between total FKQ score and other variables

Variable		*n*	*Median (IQR)*	Test *P*-value
A7. Education*	None	35	2 (3)	KW = 72⋅00 *P* < 0⋅00001 (#1)
	Primary/Secondary	531	5 (3)	
	Higher	105	7 (3)	
A11. Job^§^	None	228	4 (4)	KW = 47⋅97 *P* < 0⋅00001 (#2)
	Business	279	6 (4)	
	Food-related	109	5 (4)	
	Other	55	5 (3)	
A16. On diet	No	580	5 (4)	*U* = 2⋅18 *P* = 0⋅029
	Yes	91	5 (2)	
A17. Information Sources (Social Environment)	No	598	5 (3)	*U* = 3⋅06 *P* = 0⋅002
	Yes	73	5 (3)	
A17. Information Sources (Media)	No	429	5 (4)	*U* = −2⋅09 *P* = 0⋅037
	Yes	242	6 (3)	

KW = Kruskal–Wallis test statistic. *U* = Mann–Whitney's *U* test statistic.

* Responses collected in the questionnaires were recoded as None, Primary/Secondary and Higher.

§ Responses collected in the questionnaires (free text) were classified as None, Business, Food-related and Other.

(#1) *Post-hoc* tests: None *v*. Prim./Sec. *z* = −6⋅33, *P* < 0⋅00001; None *v*. Higher *z* = −7⋅48, *P* < 0⋅00001; Prim./Sec. *v*. Higher *z* = −5⋅31, *P* < 0⋅00001. Corrected significance threshold: 0⋅0167.

(#2) *Post-hoc* tests: None *v*. Business *z* = −6⋅70, *P* < 0⋅00001; None *v*. Food-related *z* = −2⋅06, *P* = 0⋅039; None *v*. Other *z* = −1⋅77, *P* = 0⋅076; Business *v*. Food-related *z* = 3⋅35, *P* = 0⋅00081; Business *v*. Other *z* = 2⋅91, *P* = 0⋅00361; Food-related *v*. Other *z* = −0⋅12, *P* = 0⋅908. Corrected significance threshold: 0⋅0083.

MCA was applied to disclose profiles of information sources on food and diet (item A17), leading to the identification of two distinct profiles: those who seek information in their social environment (religion, family, school, market) and those who seek information from media (fliers, television, social media) (Supplementary Figure S2). Out of the 671 respondents, 377 (56⋅2 %) did not list any source of information, while 52 (7⋅8 %) relied on their own ‘social environment’ (‘social environment profile’) and 221 (32⋅9 %) resulted in the ‘media’ profile; only 21 (3⋅1 %) were using both social environment and social media as information sources. Radio and health personnel were listed by a vast majority of respondents, so they were not fitting such profiles. The total FKQ score was slightly lower among those in the ‘social environment’ profile, and slightly higher among those in the ‘media’ profile (Table 2).

On the other hand, the total FKQ score was not related to either family's weekly money expenditure for food (A13), monthly income of the respondent (A14) or monthly income of the family (A15) (Spearman's rank correlations, data not shown).

## Discussion

This article aims to show the use of the FKQ score as a valid measure of FK for Tanzanian women of childbearing age and to describe its relation with socio-demographic characteristics. Considering the current worldwide situation, above all in developing countries, a validated FKQ could be used in future studies to identify women at higher risk of unhealthy eating habits and to tailor target-specific educational intervention with a ‘domino’ positive impact on the nutritional status and well-being of the whole society.

This article presents evidence supporting the validity of use of the FK score, computed as a total score from the FKQ. The median-referenced criterion for score computation was supported by the results of an MCA in which sections’ scores were dichotomised according to each section's median score (Fig. 1). It is noteworthy that the chosen approach for the total FKQ score is intrinsically based on a norm-referenced criterion. This means that the interpretation of the score of a subject is relative to the distribution of the score in the population: the higher the score, the more the respondent has a higher level of FK compared with its own population group. In other words, this score is an easy tool to identify those who most need educational interventions among the investigated population, like women with lower FKQ scores.

Beyond the sections measuring FK, as described elsewhere^(12)^, the FKQ includes Section A, which collects socio-demographic information including education level, job, following a diet or not. This section was designed to provide a so-called anchor test to explore the relationship between FKQ scores and several population characteristics.

The FKQ score showed a statistically significant difference between women with high and low education levels (*P* < 0⋅00001): the higher the level of education, the higher the FK score was (Table 2). This result is consistent with that of Liu *et al.*^(8)^ and Weerasekara *et al.*^(9)^, who described a similar association: the highest level of education among women was associated with an increase in food and nutrition knowledge.

Another important finding from this study is the identification of employment status (having or not a job) as a factor in increasing FK among women. In fact, women having a job had a higher FKQ score than those with no job (Table 2); furthermore, examining the types of jobs, those with business-related jobs scored higher than the rest. This result is very important, as it underlines how women with a job may have better access to knowledge about food, therefore potentially acquiring healthier dietary behaviours. This study is the first, as far as we know, to report how employment status affects FK in women of childbearing age in developing countries; however, more research on this topic will be needed to acquire more substantial evidence on the link between employment status and FK.

The FKQ score showed a bimodal distribution in our validation sample (Supplementary Figure S1): the presence of two peaks might suggest the existence of two ‘sub-populations’, one with higher FK and the other with lower FK (as classified by the FKQ score); differences in those sub-populations may be explained by different socio-demographic backgrounds, as suggested by the results concerning the relationship between FKQ score, employment status, and educational level, and also according to previous literature^(7,12)^. The authors acknowledge that such interpretation is mere speculation that cannot be properly investigated within the present study, but this topic certainly deserves further exploration in future research.

The ‘anchor test’ also investigated the sources of information used to acquire knowledge on the food-health binomial. The sources of information have been divided into two macro groups: social environment (religion, family, school, market) related and media (flyers, TV, social media) related. The result showed that people who had access to general information sources have a higher FK compared with people who did not have access to information, which underlines the importance of multidimensional approaches to improve people's FK. Food and nutrition information can be effectively transferred not only with a one-to-one approach, but also by proper dissemination activity using different tools.

The results also showed that those who obtained information from their social environment had a lower FK level than those who had access to the media, suggesting the need for educational intervention on food-health binomial in schools and in the religious environment; however, since nothing has been published on this specific topic, assumptions are merely speculative.

The median-referenced criterion for the total FKQ score could be easily adapted by administering the questionnaire and recomputing medians for sections’ scores; also, the ‘anchor test’ (Section A) could allow prompt comparison to the population for which the FKQ was originally validated. Noteworthy, evidence of validity was provided for Tanzanian women of childbearing age^(3,7)^: the use in different populations may require construct validity (and also score computation algorithms) to be reassessed, depending on how close such populations are to the original one for which the questionnaire was validated.

To sum up, the piece of research work presented here complements evidence of construct validity of the FKQ by providing an algorithm to compute a total score as a measure of FK. The strength of this tool, and of the score it returns, lies in the fact that the questionnaire has been validated^(12)^. Its reliability and validity indicate it as a trustworthy assessment tool for measurement of FK, representing a key asset to be considered along with dietary habits in specific population groups in which behaviour changes towards healthier eating habits are recommended.

## Supplementary material

For supplementary material accompanying this paper visit https://doi.org/10.1017/jns.2023.28. click here to view supplementary material
