# Supplementary material for: Food knowledge level among Tanzanian women of childbearing age: developing a score for the food knowledge questionnaire
Source: J Nutr Sci. 2023 Apr 4;12:e42. doi: 10.1017/jns.2023.28 (PMC10131052; doi:10.1017/jns.2023.28)

**Table 1. Descriptive statistics of Sections’ scores. N=671.**

| **Section** | **Median (95%CI)** | **IQR** | **Min – Max** | **n (%) above median** |
| --- | --- | --- | --- | --- |
| B | 5 (4 – 5) | 2 | 0 – 6 | 337 (50.2) |
| C | 2 (2 – 2) | 1 | 0 – 3 | 466 (69.5) |
| D | 3 (2 – 3) | 3 | 0 – 4 | 353 (52.6) |
| E | 4 (3 – 4) | 2 | 0 – 4 | 358 (53.4) |
| F | 3 (3 – 3) | 2 | 0 – 4 | 401 (59.8) |
| G | 5 (4 – 5) | 3 | 0 – 6 | 346 (51.6) |
| H | 3 (3 – 3) | 1 | 0 – 3 | 426 (63.5) |
| I | 2 (2 – 2) | 2 | 0 – 3 | 499 (74.4) |
| L | 6 (6 – 7) | 4 | 0 – 10 | 387 (57.7) |

*95%CI: Binomial exact confidence interval of the median, confidence level 95%.*

**Table 2. Association between Total FKQ score and other variables.**

| **Variable** | | ***n*** | ***Median (IQR)*** | **Test**  **p-value** |
| --- | --- | --- | --- | --- |
| **A7. Education*** | None | 35 | 2 (3) | KW=72.00  p<0.00001  (#1) |
|  | Primary/Secondary | 531 | 5 (3) |  |
|  | Higher | 105 | 7 (3) |  |
| **A11. Job^§^** | None | 228 | 4 (4) | KW=47.97  p<0.00001  (#2) |
|  | Business | 279 | 6 (4) |  |
|  | Food-related | 109 | 5 (4) |  |
|  | Other | 55 | 5 (3) |  |
| **A16. On diet** | No | 580 | 5 (4) | U=2.18 p=0.029 |
|  | Yes | 91 | 5 (2) |  |
| **A17. Information Sources (Social Environment)** | No | 598 | 5 (3) | U=3.06 p=0.002 |
|  | Yes | 73 | 5 (3) |  |
| **A17. Information Sources (Media)** | No | 429 | 5 (4) | U=-2.09 p=0.037 |
|  | Yes | 242 | 6 (3) |  |

*KW=Kruskal-Wallis test statistic. U= Mann-Whitney’s U test statistic.*

** Responses collected in the questionnaires were recoded as None, Primary/Secondary, Higher.*

*§ Responses collected in the questionnaires (free text) were classified as None, Business, Food-related, Other.*

*(#1) Post-hoc tests: None vs Prim./Sec. z=-6.33, p<0.00001; None vs Higher z=-7.48, p<0.00001; Prim./Sec. vs Higher z=-5.31, p<0.00001. Corrected significance threshold: 0.0167.*

*(#2) Post-hoc tests: None vs Business z=-6.70, p<0.00001; None vs Food-related z=-2.06, p=0.039; None vs Other z=-1.77, p=0.076; Business vs Food-related z=3.35, p=0.00081; Business vs Other z=2.91, p=0.00361; Food-related vs Other z=-0.12, p=0.908. Corrected significance threshold: 0.0083*

**Figure 1. Coordinates Plot (Plot A) and Projections Plot (Plot B) of the Multiple Correspondence Analysis on dichotomised Sections’ scores. Dimension 1 (horizontal axis in Plot A) explains 90.2% of inertia, i.e. observed variability. Coordinates are reported in principal normalization.**


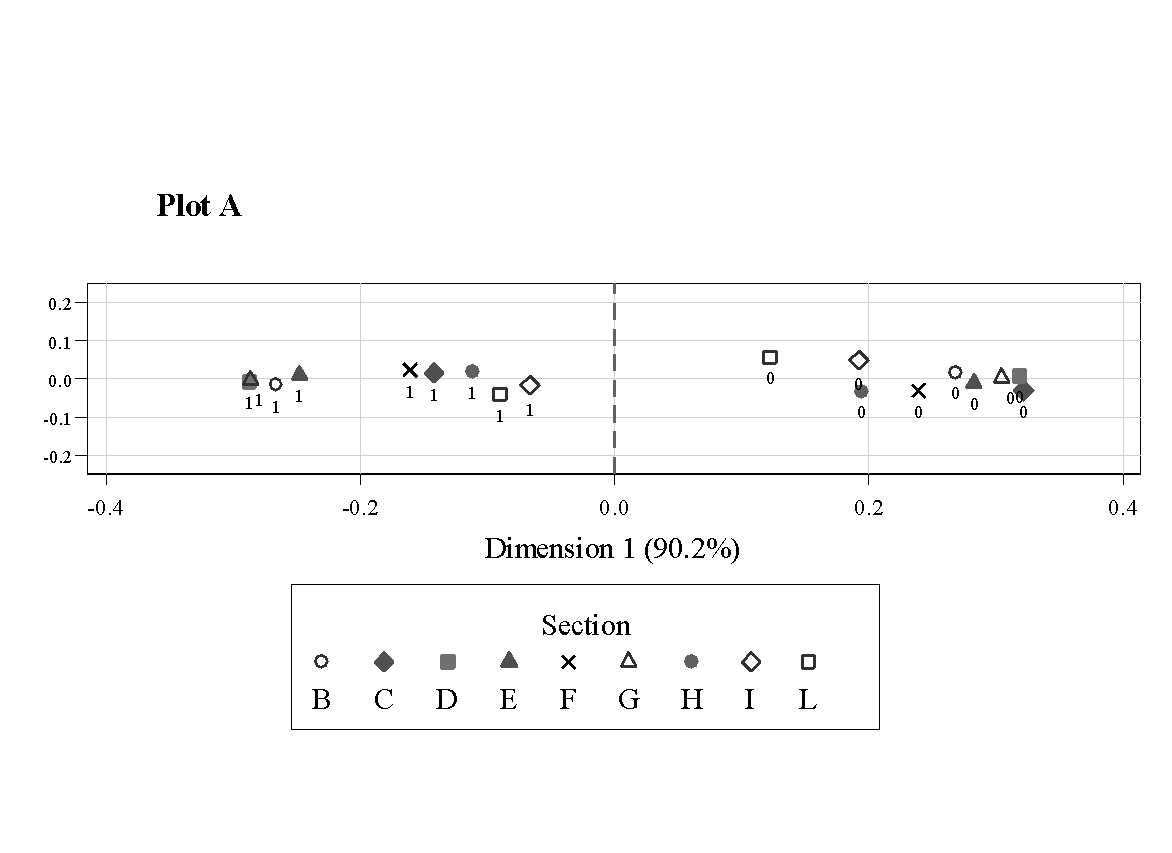


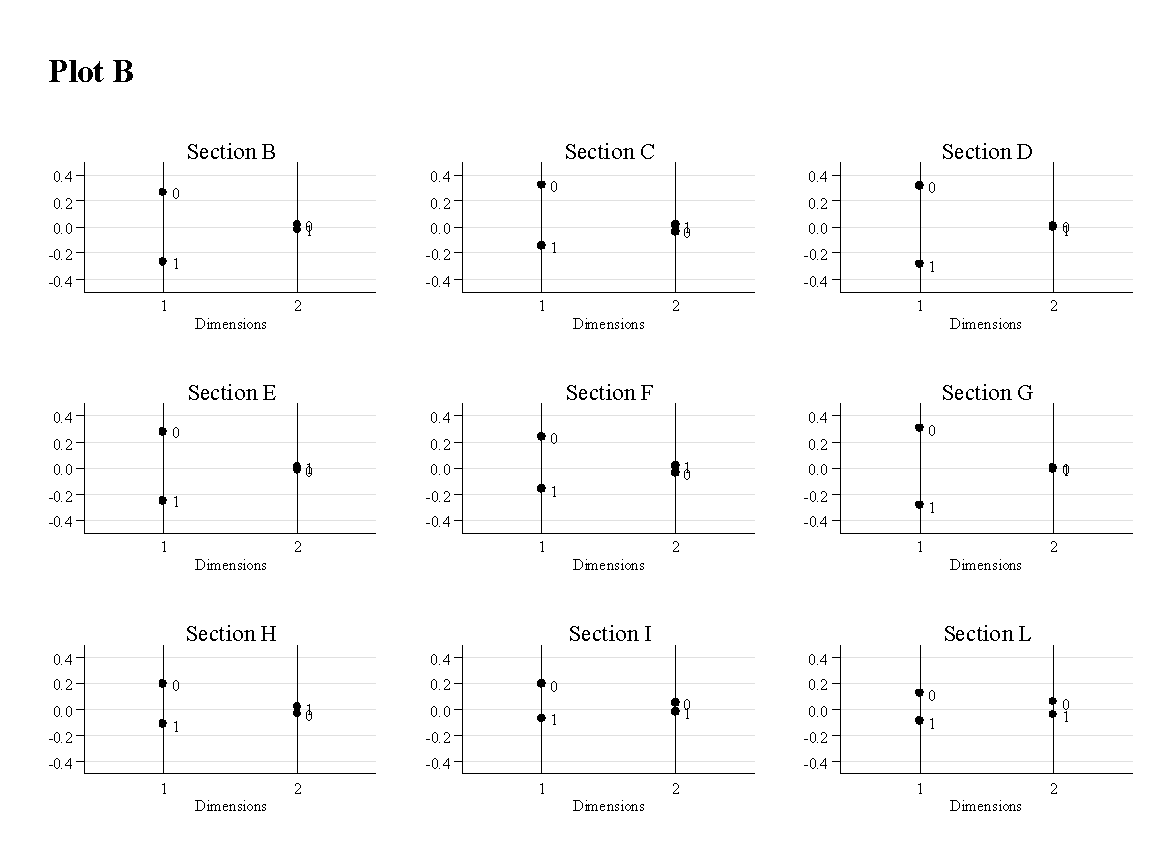

Supplement: Supplementary file 1 [file jnssup.zip › S2048679023000289sup001.docx]
