# Supplementary material for: Food knowledge level among Tanzanian women of childbearing age: developing a score for the food knowledge questionnaire
Source: J Nutr Sci. 2023 Apr 4;12:e42. doi: 10.1017/jns.2023.28 (PMC10131052; doi:10.1017/jns.2023.28)
Supplement: Supplementary file 1 [file jnssup.zip › S2048679023000289sup002.docx]

**Food Knowledge level among Tanzanian women of childbearing age: developing a score for the Food Knowledge Questionnaire**

Maria Vittoria Conti 1*, Marco Gnesi 2*, Naelijwa Mshanga 3, Rachele De Giuseppe 1, Francesca Giampieri 4,5, Hellas Cena 1,6

1. Laboratory of Dietetics and Clinical Nutrition, Department of Public Health, Experimental and Forensic Medicine, University of Pavia, Via Bassi 21, 27100 Pavia, Italy;
2. Section of Biostatistics and Clinical Epidemiology, Department of Public Health, Experimental and Forensic Medicine, University of Pavia, 27100 Pavia, Italy;
3. The Nelson Mandela African Institution of Science and Technology, Arusha 447, Tanzania;
4. Department of Biochemistry, Faculty of Sciences, King Abdulaziz University, Jeddah 21589, Saudi Arabia 10 4
5. Research Group on Food, Nutritional Biochemistry and Health, Universidad Europea del Atlantico, Santan 11 der, 39011, Spain
6. Clinical Nutrition and Dietetics Service, Unit of Internal Medicine and Endocrinology, ICS Maugeri IRCCS, 27100 Pavia, Italy

**Corresponding author:**

Maria Vittoria Conti

Via bassi 21 Pavia

[mariavittoria.conti@unipv.i](mailto:mariavittoria.conti@unipv.ir)

[0382987536](tel:0382987536)

**Short title:** Validating a FKQ score for Tanzanian women

**Acknowledgements:** None

**Financial Support:** This manuscript was realized in the context of a cross-sectional study within the Sustainable Agri-Food System Strategies (SASS) project, funded by the Italian Ministry of University and Research (MIUR) (Fondo integrativo speciale per la ricerca, FISR; CUP: H42F16002450001).

**Conflict of Interest:** Marco Gnesi is current employee at AstraZeneca. His current employee had no role in designing, conducting, analysing and interpreting the research work presented in this manuscript.

**Authorship:**  Conceptualization MVC, MG, RDG and HC; methodology MVC, MG.; software MVC; validation MVC, MG; formal analysis MG; investigation NM; resources NM; data curation NM, MVC; writing—original draft preparation MVC, MG and RDG; writing—review and editing MVC, MG, FG, RG; visualization FG; supervision HC; project administration MVC; funding acquisition HC. All authors have read and agreed to the published version of the manuscript.

**Ethical Standards Disclosure:** This study was conducted according to the guidelines laid down in the Declaration of Helsinki and all procedures involving research study participants were approved by the DPRTC/R/ 142 Val. II/ 10. Written informed consent was obtained from all subjects/patients. Study approval was granted by the Sokoyne University of Agriculture (SUA), located in Morogoro city (Tanzania). To facilitate research, the Vice-Chancellor of the SUA was empowered to issue research clearance to staff, students, research associates and researchers of SUA on behalf of the Tanzania Commission for Science and Technology within the Sustainable Agri-Food Systems Strategies project, funded by the Italian Ministry of University and Research (MIUR) (Fondo integrativo speciale per la ricerca, FISR; CUP: H42F16002450001).
